# Immunoregulatory Effects of Bone Marrow-Derived Mesenchymal Stem Cells in the Nasal Polyp Microenvironment

**DOI:** 10.1155/2014/583409

**Published:** 2014-02-13

**Authors:** Rogério Pezato, Danilo Cândido de Almeida, Thiago Freire Bezerra, Fernando de Sá Silva, Claudina Perez-Novo, Luís Carlos Gregório, Richard Louis Voegels, Niels Olsen Câmara, Claus Bachert

**Affiliations:** ^1^Upper Airway Research Laboratory (URL), Department of Otorhinolaryngology, Ghent University Hospital, Ghent University, 9000 Ghent, Belgium; ^2^Department of Otolaryngology-Head and Neck Surgery, Federal University of São Paulo, Rua dos Otonis, 700 Piso Superior, Vila Clementino, 04025-002 São Paulo, SP, Brazil; ^3^Nephrology Division, Department of Medicine, Federal University of São Paulo, 04025-002 São Paulo, SP, Brazil; ^4^Department of Otorhinolaryngology and Ophthalmology, University of São Paulo, 05508-070 São Paulo, SP, Brazil; ^5^Department of Immunology, Institute of Biomedical Sciences IV, University of São Paulo, 05508-070 São Paulo, SP, Brazil

## Abstract

Nasal polyposis is a severe, chronic inflammatory condition of the paranasal sinuses and is frequently associated with asthma and aspirin sensitivity. Mesenchymal stem cells exhibit a potent immunosuppressive effect in several inflammatory conditions, and their role in nasal polyposis remains little explored. Hence, we investigated whether bone marrow-derived mesenchymal stem cells could modulate cell phenotype in the nasal polyp milieu. After coculture with mesenchymal stem cells, the frequency of these inflammatory cells was found to decrease. Furthermore, mesenchymal stem cells promoted strong inhibition of CD4+ and CD8+ T cell proliferation, increased the frequency of CD4+CD25+Foxp3 T cells, and changed the global cytokine profile from an inflammatory to an anti-inflammatory response. We believe that mesenchymal stem cells may be a very useful adjunct for investigation of the inflammatory process in nasal polyposis, contributing to better understanding of the inflammatory course of this condition.

## 1. Introduction

Nasal polyposis (NP) is a severe, chronic inflammatory condition of the paranasal sinuses, with a prevalence ranging from 1% to 4% in the general population [1]. It is frequently associated with asthma and aspirin sensitivity [1]. NP in combination with aspirin-induced asthma (AIA) represents the most severe form of airway inflammation [2].

NP is characterized by overgrowth of nasal mucosa caused by the influx of a variety of inflammatory cells. The inflammatory process characteristic of NP is defined mainly by T-cell activation and arrest of regulatory T-cell function, with a decrease in Foxp3 expression and concomitant upregulation of T-bet and GATA-3 levels [3]. The predominance of T-effector cells in polyp tissue is closely associated with patient ethnicity. In white European patients a Th2-driven response is predominant, whereas in Chinese patients, a Th1/Th17-driven response has been demonstrated [4]. However, little is known about the inflammatory milieu of nasal polyposis, and understanding of this process can play an important role in defining the course of the disease.

The most important features of NP concern its unique remodeling process, which is characterized by low production of transforming growth factor-*β* (TGF-*β*) associated with a lack of collagen as compared with healthy subjects [5, 6]. In NP, matrix metalloprotease-7 (MMP-7) and matrix metalloprotease-9 (MMP-9) levels are increased and tissue inhibitor of metalloproteinases 1 (TIMP-1) levels are, inversely, decreased as compared with normal nasal mucosa [7]. This imbalance can be partially explained by the lack of TGF-*β*1 in NP and its inhibitory effect on MMP-9 activity via TIMP-1 release [8]. TGF-*β*1-activated PAI-1 (plasminogen activator inhibitor-1) is also found decreased in NP, leading to an increase in the levels of plasminogen activator and MMP levels when compared with controls [6, 9].

Due to the similarities between upper and lower airway mucosa, the last one has been used as a model to understand NP [10]. It is demonstrated an epithelium disruption in nasal tissue less extensive than in the lungs [11], so to repair the injured epithelium less TGF-*β* is produced in nasal mucosa when compared to bronchial mucosa [12]. Supporting these findings it is reported that the basement membrane in nasal mucosa has limited pseudothickening with significant less elastase positive cells comparatively to bronchial mucosa [11].

Furthermore, histological examination of biopsy specimens shows a soft tissue with clear lack of extracellular matrix [13], major edema, albumin-filled pseudocysts, and alpha-2-macroglobulin [14].

Multipotent stromal cells or mesenchymal stem cells (MSC) are adult, adherent, nonhematopoietic stem cells with the ability to differentiate into several mesenchymal cell lines (chondrocytes, adipocytes, and osteocytes) beyond to promote a prominent immunomodulatory effects on inflamed environment. MSCs retain low immunogenicity and exert immunosuppressive effects in allogeneic transplantation [15]. These cells exhibit reduced expression of both major histocompatibility complex (MHCs) and costimulatory molecules (CD80, CD86, and CD40) and have emerged as a very useful tool for therapeutic use, including in regenerative medicine and tissue bioengineering [16, 17].

MSCs have been used in experiments involving a very broad range of diseases, including repair of acutely injured tissue, chronic diseases, graft rejection, and autoimmune conditions [18]. Such widespread use of these cells is based on their distinct natural properties, namely, stromal cell differentiation, soluble factor secretion stimulating hematopoiesis, ECM maintenance, and immunoregulatory effects [19]. The immunomodulatory role of MSCs has been demonstrated in many *in vitro* and *in vivo* studies and consists essentially of downmodulation of the inflammatory process, inhibiting T-cell, B cell, NK cell, and APC cell proliferation via a paracrine secretory mechanism [20–22].

Most soluble factors produced by MSCs are associated with their immunoregulatory properties, including TGF-*β*1, prostaglandin-E2 (PGE-2), IDO, IL-10, IL-6, MMP, and TIMP [17], and could interact with severely inflamed tissues (such as the NP environment) to restore a balanced T cell-mediated response. Hence, we hypothesized that MSCs could be able to modulate allergic inflammation in the context of nasal polyposis.

Using MSCs as an immunosuppressive tool, we first characterized the infiltrating polyp-derived cells and found that, after coculture with MSCs, the frequency and activation of inflammatory cells had changed. We also observed that MSC promoted modulation of the cytokine profile, inhibition of T-cell proliferation, and expansion of CD4+CD25+Foxp3 cells in an *in vitro* assay.

## 2. Material and Methods

### 2.1. Patients and Clinical Diagnosis

Nasal polyp tissue samples of 12 patients with known NP were obtained during functional endoscopic sinus surgery (FESS) performed at the Department of Otorhinolaryngology, University of São Paulo, Brazil. The study was approved by the local Research Ethics Committee and written informed consent was obtained from each patient before sample collection. The diagnosis of NP was based on medical history, clinical examination, nasal endoscopy, and computed tomography (CT) of the paranasal sinuses according to the European Position Paper on the Primary Care Diagnosis and Management of Rhinosinusitis and Nasal Polyps 2012 [23].

All subjects underwent a skin prick test for common inhalant allergens. The diagnosis of asthma was obtained from the Department of Pulmonology at the University of São Paulo. Information on aspirin intolerance was collected from patient histories (Table 1).

### 2.2. MSC Isolation and Preparation of Nasal Polyp Single-Cell Suspension

Human mesenchymal stem cells (MSCs) were isolated by rinsing of the cells remaining in bone marrow collection tubes, kindly provided by the Children's Hospital of São Paulo and GRAACC (Support Group for Children and Adolescents with Cancer), with ethical approval and the informed consent of the donors (Protocol no. 45/09). The tubes were washed with PBS, cells were isolated by the Ficoll-Hypaque protocol (Sigma, USA), and culture was performed as previously described by Lennon and Caplan (2006) and Pittenger et al. (1999) [24, 25]. The cells were cultured in basal medium containing DMEM-low glucose medium (Gibco, USA) supplemented with 10% HyClone fetal bovine serum (Thermo, USA), antibiotic (100 U/mL penicillin and 100 *μ*g/mL streptomycin, Gibco), and 100 mM of nonessential amino acids (Gibco). The MSCs were subcultured for 10 passages and then used for coculture experiments with polyp-derived cells. All polyp-derived cells were isolated from polyp tissues by mechanical separation (with surgical scissors and forceps) followed by digestion in collagenase IV (1 mg/mL, Sigma) for 50 minutes at 37°C. The cells were then washed in complete medium (10% serum), filtered in a 70 *μ*m cell strainer (BD Biosciences, USA), suspended, and seeded onto six well plates containing R10 medium: RPMI-1640 culture medium (Gibco), supplemented with 10% FBS (Gibco) and antibiotic (100 U/mL penicillin and 100 *μ*g/mL streptomycin; Gibco). During coculture assays, both MSCs and polyp-derived cells were incubated at 37°C in a 5% CO_2_ atmosphere for 72 hours.

### 2.3. Phenotype and Differentiation Capacity of MSCs

To characterize MSC phenotype, the cells were harvested by treatment with trypsin (Gibco), washed, and suspended in phosphate-buffered saline (PBS), and approximately 1 × 10^5^ cells were incubated with conjugated monoclonal antibodies (1 : 100) against CD73, CD90, CD105, CD54, CD45, HLA-DR, PDL-1, PDL-2, and CTLA-4 (conjugated with PE, FITC, or PerCP fluorochrome; BD Biosciences). The FACSCanto II flow cytometer (BD, Biosciences) was used for acquisition, and analysis was performed using the FlowJo software, version 7.2.4 (Tree Star, USA). To assess the ability to differentiate into several mesenchymal cell lines, the cells were subjected to adipogenic, chondrogenic, and osteogenic differentiation protocols, as described by Pittenger et al., 1999 [25]. The stem cells were cultivated at 95% confluence and multipotential differentiation was induced with specific agents using the Mesenchymal Stem Cell Adipogenesis, Osteogenesis, and Chondrogenesis Kit, according to manufacturer instructions (Chemicon, USA). The medium was replaced every 4 days for 21 days. Adipocyte-like cells were stained with Oil Red O to assess neutral lipid accumulation in fat vacuoles. Chondrocyte-like cells were stained using Safranin O, and osteogenesis specimen cells were stained with Alizarin Red for intracellular calcium compounds.

### 2.4. Cellular Phenotyping of Nasal Polyp-Derived Tissue

To determine the immune cell profile of whole polyp cells, fresh and cultured (for 72 hours) cells were harvested (both adherent and nonadherent cells) without enzymatic dissociation and labeled with a set of various specific fluorescent antibodies, such as CD4-Pacifc Blue, CD8-PECy7, CD11c-PE, CD14-PerCPCy5.5, CD19-Viollet, CD25-APC, CD40-FITC, CD69-FITC, and NK1.1-PE (all from BD Biosciences). These sets of antibodies were used for both fresh and *in vitro* coculture analysis. To investigate the CD4+CD25+Foxp3+ T lymphocyte profile, intracellular staining for FoxP3 expression was performed after 72 hours in culture with or without the presence of MSCs. The cells were fixed and permeabilized using the Fix/Perm Buffer Set Kit (BD Biosciences). Staining was performed using FoxP3 (PE), CD4 (APC-Cy7), and CD25 (APC) antibodies at 1 : 100 dilution (BD Biosciences). Analysis was determined by flow cytometry (FACSCanto II cytometer, BD Biosciences) within FSC and SSC gate in CD4+ T cells. All acquisitions were performed using a FACSCanto II flow cytometer (BD Biosciences), and analysis was again done using the FlowJo 7.2.4 software (Tree Star). During analysis, gates for mononuclear cells were performed in FSC and SSC parameters after doublet exclusion. Results are presented as cell frequency.

### 2.5. T-Cell Proliferation Assay

To determine the ability of MSCs to modulate the polyp microenvironment, fresh whole polyp cells were labeled with 5 *μ*M carboxyfluorescein succinimidyl ester (CFSE, BD Bioscience) and seeded in culture with or without MSCs for 5 days. MSCs from nonrelated donors were cocultured with 1 × 10^5^ whole polyp cells in a 1 : 3 ratio in R10 medium supplemented with 100 mM of vitamin complex (Thermo), antibiotic (Pen/Strep, 100 U/mL, Gibco), 100 mM L-Glutamine Mixture (Gibco), 100 mM MEM nonessential amino acids (Gibco), and 1 mM HyClone sodium pyruvate (Thermo). After 5 days, all polyp cells were stained with anti-CD4 and anti-CD8 antibodies (1 : 100 ratio, BD Biosciences), and CFSE dilution was read in FITC channel by flow cytometry analysis (FACSCanto II cytometer, BD Biosciences). This analysis was performed within the FSC and SSC gate using histograms for the T-cell type of interest (CD4+ or CD8+). Phytohemagglutinin A (1 *μ*g/mL) (PHA, Invitrogen, USA) was used as a polyclonal-positive stimulus.

### 2.6. Cytokine Profile of Nasal Polyp-Derived Cells

Isolated polyp culture and coculture (MSC and polyps) supernatants (from 6 out of 12 NP samples) were further harvested after 72 hours for cytokine quantification, using the Cytometric Bead Array (CBA) Th1/Th2/Th17 Cytokine Kit, according to manufacturer recommendations (BD, Becton Dickinson Biosciences). Acquisition was performed in the FACSCanto II cytometer (BD Biosciences) and the samples analyzed using FCAP Array software v3.0 (Soft Flow Inc., HUN).

### 2.7. Statistical Analysis

Results were assessed for normal distribution by the Kolmogorov-Smirnov test. Categorical variables were expressed as percentages (%), and continuous variables (data) were presented as means ± standard deviations. The Mann-Whitney nonparametric test was used to assess between-group differences. For analysis among three or more groups, the Kruskal-Wallis nonparametric test for one-way analysis of variance was used. For all analyses, a *P* value ≤ 0.05 was considered statistically significant. All statistical testing and plotting were performed using GraphPad Prism 5 (GraphPad software, Inc., USA).

## 3. Results

### 3.1. Characterization of Mesenchymal Stem Cells and Polyp Infiltrating Cells

**Figure 1:**
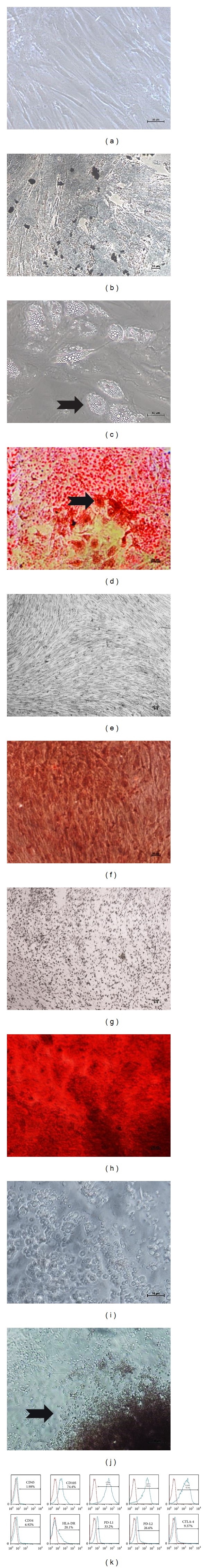
Characterization of bone marrow-derived mesenchymal stem cells and polyp-derived cells. (a) MSCs exhibiting fibroblastic-like morphology *in vitro*; (b) MSC culture in adipogenic control medium showing absence of lipid vesicles; (c) MSCs containing lipid vesicles (black arrow) in adipogenic differentiation medium without Oil Red stain; (d) MSC culture in adipogenic differentiation medium stained with Oil Red (black arrow); (e) MSC culture in chondrogenic control medium; (f) MSC culture in chondrogenic differentiation medium stained with Safranin O; (g) MSC culture in osteocyte control medium; (h) MSC culture in osteogenic differentiation medium stained with Alizarin Red; ((i)-(j)) representative view of polyp-derived cells spreading and growing in culture (black arrow); and (k) immunophenotypic signature of MSCs in culture, demonstrating absence of hematopoietic (CD45 and CD34) and immunogenic (HLA-DR) markers and expression of CD105, CD90, CD73, and CD54, as well as immunoregulatory receptors such as PDL-1 and -2 and CTLA-4.

The bone marrow-derived mesenchymal stem cells used in this study exhibited most important characteristics of MSCs, such as a plastic-adherent growth pattern (Figure 1), fibroblast-like morphology (Figure 1(a)), expression of specific surface antigens (CD105, CD54, CD90, and CD73), and immunoregulatory molecules (CTLA-4, PD-L1, and PD-L2), and no expression of the immunogenic and hematopoietic surface markers HLA-DR and CD45, respectively (Figure 1(k)). Additionally, MSCs demonstrated the ability to differentiate into adipocytes (Figures 1(c)-1(d)), chondrocytes (Figure 1(f)), and osteocytes (Figure 1(h)) *in vitro*. The polyp-derived cells spread from the whole polyp in culture and exhibited spheroid-like morphology (Figures 1(i) and 1(j)).

**Figure 2:**
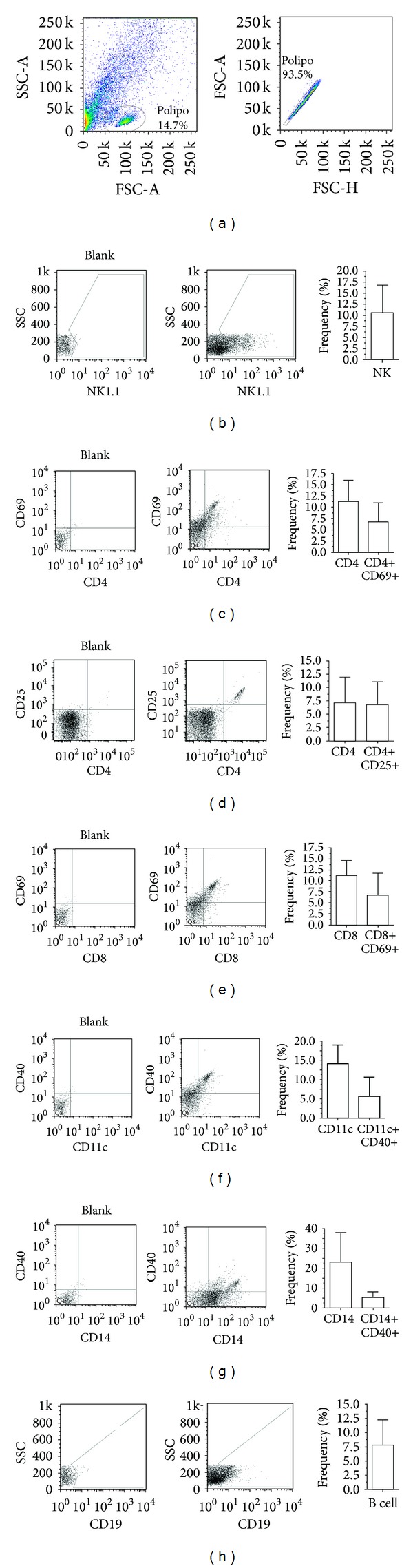
Phenotypic aspects of polyp-derived cells. (a) Gate strategies for selection of the mononuclear fraction of polyp-derived cells; (b) NK cells (NK1.1+); (c) CD4 and CD4+CD69+ T cells; (d) CD4+CD25+ T cells; (e) CD8 and CD8+CD69+ T cells; (f) CD11c+ and CD11c+CD40+ dendritic cells; (g) CD14+ and CD14+CD40+ monocytes and (h) B cells (CD19+). Several types of activated and nonactivated immune cells were observed within the polyp parenchyma.

The immunophenotype of whole NP-derived cells showed the presence of distinct immune cells after single-cell suspension. NK cells, T cells, dendritic cells, monocytes, and B cells were present (Figures 2(a)–2(h)). The frequency of each subtype of immune cells was as follows: NK cells, 10.60 ± 6.28%; CD4+, 11.26 ± 4.70%; CD4+CD69+, 6.71 ± 4.31%; CD4+CD25+, 6.77 ± 4.35%; CD8+, 11.19 ± 3.49%; CD8+CD69+, 6.73 ± 5.09%; CD11c+, 14.17 ± 4.83%; CD11c+CD40+, 5.52 ± 5.14%; CD14+, 22.88 ± 15.06%; CD14+CD40+, 5.06 ± 2.92%; and CD19+, 7.88 ± 4.36% (Figures 2(a)–2(h)). In our samples, the ratio of activated cells to nonactivated cells within the polyp ranged from 60% to 20%, depending on cell type. For CD4+ and CD8+ T cells, this index was approximately 60%, for dendritic cells 35%, and for monocytes 20% (Figures 2(a)–2(h)).

### 3.2. Effect of MSCs on Nasal Polyp-Derived Cells

**Figure 3:**
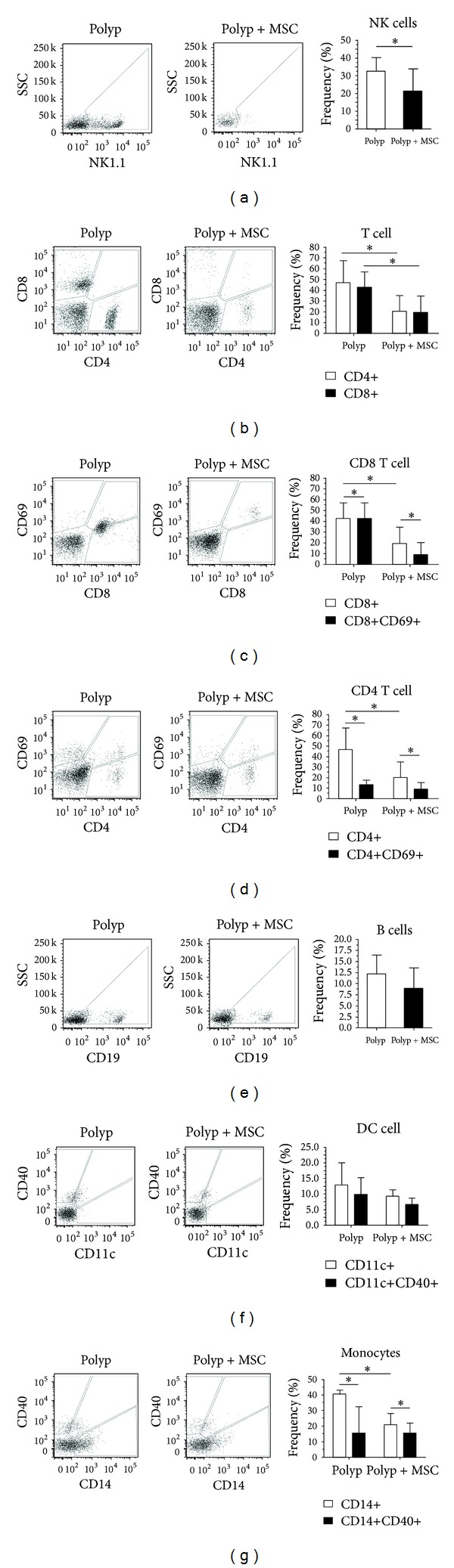
Bone marrow-derived mesenchymal stem cells modulating the polyp microenvironment *in vitro*. (a) NK cell (NK1.1+) frequency alone and in coculture with MSCs; (b) CD4+ and CD8+ frequencies alone and in coculture with MSCs; (c) CD8+ and CD69+ frequencies alone and in coculture with MSCs; (d) CD4+ and CD69+ frequencies alone and in coculture with MSCs; (e) B cell (CD19+) frequency alone and in coculture with MSCs; (f) CD11c+ and CD11c+CD40+ dendritic cell frequencies alone and in coculture with MSCs and CD14+ and CD14+CD40+ monocyte frequencies alone and in coculture with MSCs. The presence of MSCs may immunomodulate the phenotype of polyp-derived cells toward an immunosuppressive profile.

To evaluate the role of MSCs in the modulation of the NP microenvironment, we carried out a coculture assay for 72 hours and assessed the frequency of polyp-derived infiltrating cells. We found a significant decrease in the frequency of most inflammatory cells. NK cells (NK1.1+), T helper cells (CD4+), and cytotoxic T cells (CD8+) showed a significant decrease in frequency when cocultured with MSC; however, no changes in the activated T cell compartment (CD4+CD69+ and CD8+CD69+) were observed (Figures 3(a)–3(d)). The frequency of B cells, monocytes, and dendritic cells also tended to decrease after coculture with MSCs. *De novo*, the fraction of activated cells included in the monocyte and dendritic cell subpopulations was not altered in comparison with cultures performed in the absence of MSCs (Figures 3(e)–3(g)).

### 3.3. Effect of MSCs on the Expansion/Proliferation Capacity of the T-Cell Compartment in Nasal Polyp-Derived Cells

**Figure 4:**
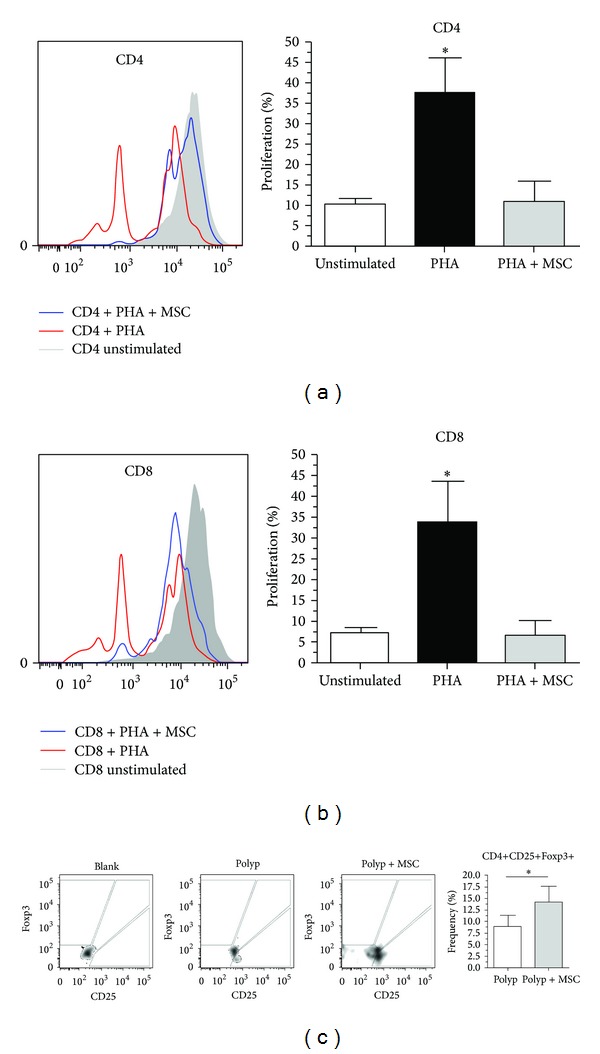
Bone marrow-derived mesenchymal stem cells exhibit functional immunosuppressive action on polyp-derived cells *in vitro*. (a, b) Proliferative index of CD4+ and CD8+ cells, respectively, stimulated with phytohemagglutinin (PHA), in the presence or absence of MSCs; (c) frequency of CD4+CD25+Fpxp3+ cells in the presence or absence of MSCs. Functionally, MSCs exhibited immunosuppressive action on polyp-derived cells, as represented by inhibition of proliferation of CD4+ and CD8+ cells with a concomitant increase in CD4+CD25+Fpxp3+ frequency.

On the basis of the fact that MSC had promoted a decrease in the frequency of most inflammatory cells in NP-derived tissue, we sought to investigate whether MSCs could promote the functional ability to arrest NP-derived T-cell proliferation *in vitro*. Surprisingly, we observed significant inhibition of expansion/proliferation of both T helper cells (CD4+) and cytotoxic T cells (CD8+) derived from NP stimulated with PHA in compared with cells stimulated but cultured in the absence of MSCs (Figures 4(a) and 4(b)). The immunosuppressive effect of MSCs on NP-derived T cells was evident, considering that the index of proliferation of CD4+ and CD8+ T cells cocultured with MSCs did not differ from that of control unstimulated T cells.

The immunoregulatory effect of MSCs is almost always associated with an expansion of regulatory T cells (CD4+CD25+Fosp3+). Thus, in the present work, we investigated the presence of CD4+CD25+Foxp3+ T cells in culture with or without the presence of MSCs. A significant increase in number of CD4+CD25+Foxp3+ T cells was observed in the presence of MSCs as compared with cultures of T cells alone (Figure 4(c)), suggesting that these cells can play an essential role in the development of the chronic inflammatory process in NP.

### 3.4. Effect of MSCs on the Cytokine Profile of Nasal Polyp-Derived Cells

**Figure 5:**
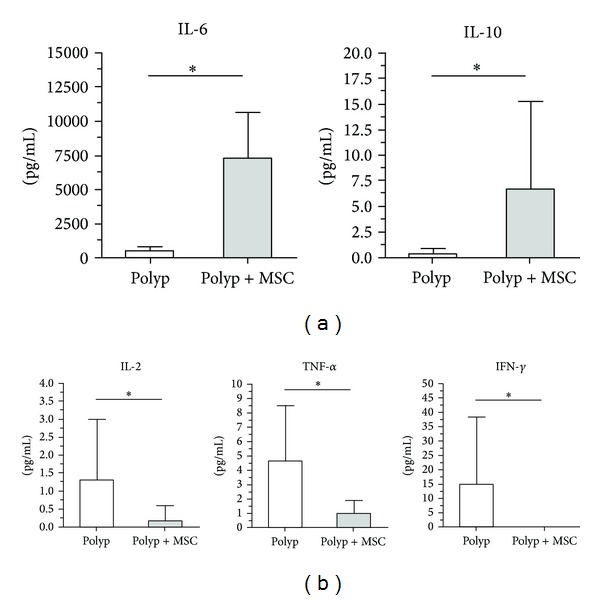
*In vitro* cytokine profile of polyp-derived cells cultured in the presence or absence of bone marrow-derived mesenchymal stem cells. A decrease in inflammatory cytokines (IL-2, TNF-*α*, and IFN-*γ*) and enhancement of anti-inflammatory cytokine (IL-10 and IL-6) levels was observed when MSCs were cocultured with polyp-derived cells.

Finally, to elucidate the mechanism underlying the immunosuppressive effect of MSCs in NP, we analyzed six NP samples using the Cytometric Bead Array Technique. Analysis of culture supernatants showed a prominent shift from an inflammatory to an anti-inflammatory cytokine profile. In coculture, MSCs promoted an increase in anti-inflammatory molecules such as IL-10, with a concomitant decrease in inflammatory cytokines such as IL-2, TNF-*α*, and IFN-*γ* (Figure 5).

## 4. Discussion

Nasal polyposis is characterized by the most severe upper-airway inflammatory process observed in clinical practice. This process is crucial to understand the mechanisms that underlie development of polyposis. It is known that MSCs have immunomodulatory properties, as demonstrated in some organs such as the brain, kidney, heart, bone, and lung [26–30]. MSC-mediated immunomodulation can occur via cell-to-cell contact or by release of soluble factors, which are associated with many regulatory effects of these cells in a tissue inflammation context. The best documented MSC-secreted cytokines are TGF-*β*1, PGE-2, IDO, IL-10, IL-6, MMP-2,9, TIMP-2,3, nitric oxide (NO), chemokine ligand 2 and 5 (CCL2,5), human leukocyte antigen 5 (HLA-G5), heme oxygenase-1 (HO-1), hepatocyte growth factor (HGF), and leukemia inhibitory factor (LIF) [17]. On the basis of their properties, MSCs have been explored in a wide range of experiments and have been used for therapeutic purposes more extensively than any other subtype of stem cell. These cells also retain further important features, such as low immunogenicity, and promote inhibition of proliferation/activation in allogeneic lymphocytes [18].

Thus, in the present study, we assessed the impact of MSCs on modulation of the inflamed NP microenvironment. Firstly, we demonstrated that several types of inflammatory cells (NK cells, B cells, T cells, monocytes, and dendritic cells) are found in this milieu. After characterization of MSC phenotype and differentiation, these cells were cultured with polyp-derived cells, and a direct immunomodulatory effect on the inflammatory NP cell compartment was observed. There was a significant decrease in the frequency of CD4+, CD8+, CD14+, and NK cells, as well as a significant increase in CD4+CD25+Foxp3+ T cells, when MSCs were cocultured with NP-derived cells. A decrease in regulatory T cells has been described as a feature of the NP disease course. This is an important finding, and the increase in CD4+CD25+Foxp3 cells induced by presence of MSCs in the present study may help in our understanding of the progression of NP. Functionally, we observed that MSCs inhibited both CD4+ and CD8+ polyp-derived T cell proliferation and efficiently changed the local inflammatory pattern, promoting a shift from an inflammatory to an anti-inflammatory cytokine profile.

The stem cells actions are widely dependent on the disease and time of therapy. Contact-dependent immunosuppression is one mechanism of MSC action and can be associated with the expression of immunoregulatory molecules expressed on the surface of MSCs or by delivering microvesicles carrying bioactive molecules [31]. CTLA-4, PDL-1, and PDL-2 were present in the MSCs used in this study, and their role in inhibition of proliferation and activation of T lymphocytes has been reported extensively in the stem cell literature [32, 33].

MSC is also capable of exerting influence on inflamed process via paracrine action [34] even far from the injured tissue. The soluble factors released by MSCs are another mechanism that may also have influenced modulation of the polyp-derived cell microenvironment. Many studies have demonstrated the effect of TGF-*β* secreted by MSCs on suppression of peripheral blood mononuclear cell (PBMC) proliferation [35]. TGF-*β* is also capable of increasing the frequency of regulatory T cells, especially when associated with PGE2 [36–38]. In a model of asthmatic mice, TGF-*β* secreted by MSCs showed a beneficial effect by decreasing levels of IL-4, IL-5, IL-13, and immunoglobulin in bronchoalveolar lavage fluid [38].

Although the role of TGF-*β* on remodeling process is still controversial in the literature, there is a strong evidence in NP that the lack of TGF-*β* is involved in decreasing extracellular matrix formation [5, 6, 39] highlighting the importance of this molecule on the development of inflammation in NP.

In addition, PGE2 is another molecule which is found in low levels in NP tissue [40] and MSCs produce PGE2. PGE2 exerts an immunosuppressive action on T cells and, consequently, promotes a decrease in IFN-*γ* and TNF-*α* levels [41, 42]. Our study corroborates these findings, demonstrating decreased IFN-*γ* and TNF-*α* expression in NP cells cocultured with MSCs in comparison with cultures of NP-derived cells alone; maybe PGE2 is contributing to decrease these interleukins.

In mice, macrophages from septic lungs produced higher levels of IL-10 when treated with MSCs than untreated macrophages. The authors suggested that the EP2 and EP4 receptors (prostaglandin receptors) were responsible for this increase in IL-10 production [43]. In this context, IL-10 is considered to be the main immunosuppressive interleukin, and we found higher levels of IL-10 in NP cells treated with MSCs than in untreated cells.

Consistently with what is found in a variety of other diseases [44, 45], the presence of MSCs in NP cell cultures increased the expression of IL-6 (an interleukin mainly produced by Th1 cells). Paradoxically, although IL-6 is associated with increased production of IL-2 [46], we found decreased levels of this inflammatory interleukin in NP cells treated with MSCs. One plausible explanation is the fact that MSCs can secrete TGF-*β* and IL-6 and that differentiation of Treg/Th17 depends on the proportion of these two cytokines [47]. In the present study, we did not detect IL-17 production in NP cell cultures, with or without the presence of MSCs. This may suggest that the interaction of distinct soluble factors with different cell types could alter the immune context of NP.

Furthermore, indoleamine 2,3-dioxygenase (IDO), a rate-limiting enzyme that catalyzes the degradation of tryptophan, is found at increased levels during chronic inflammatory diseases induced by inflammatory mediators such as IFNs and IL-6 [48]. Elevated IDO expression has been observed in nasal polypoid tissue as compared with healthy nasal mucosa [49]. IDO is an immunoregulatory enzyme and belongs to the MSC arsenal. The role of MSCs in induction of tolerance in renal allograft recipients was not confirmed in IDO knockout mice, showing the crucial importance of IDO to the immunosuppressive effect of MSCs via regulatory T cells [50].

In addition, we observed an elevated presence of IFN-*γ* and IL-6 in cultures of NP-derived cells. After coculture with MSCs, IL-6 levels increased whereas IFN-*γ* declined. It is important to note that a decrease in IFN-*γ* could indicate a decrease in Th1 cells activation.

Different types of T cells are implicated in the pathogenesis and progression of NP, but, in populations of European descent, Th2-driven disease is still a hallmark of the condition. In the Asian population NP is a Th17/Th1-driven disease with increase of neutrophils. We characterize our patients as descendants of European and none was of Asian origin. In our sample the inflammatory cells profile was similar to the one found in European population (increase of Th2 cells and eosinophils).

Some studies have demonstrated that MSCs are able to promote conversion of the Th1 phenotype into a Th2 response [51]. In this sense, MSCs could contribute to NP, considering the microenvironment already saturated with Th2-dependent interleukins in this setting. In the present study, we did not detect IL-4 which is considered an important interleukin that induces differentiation from Th0 to Th2 immune response in NP-derived cell cultures, regardless of the presence of MSCs. This would suggest that in our coculture experiments with NP-derived cells, MSCs could not intensify a specific Th2 response.

Additionally, in the presence of MSCs, Th1 cytokines profile was altered, Th2 cytokines were not detected, and IL-10 was increased. These results indirectly suggest that the T-cell profile may have been directed to a regulatory pattern, considering the prominent increase of CD4+CD25+Foxp3+ T cell frequency in our MSC cocultures.

The NP treatment is based on two main pillars: oral and topical steroids and surgery, with the recurrence of NP after surgery being usual. The understanding of MSC mechanism in decreasing the inflammatory process in NP could be helpful to reduce the intake of steroids and the surgery indications.

In conclusion, we demonstrated that MSCs can be a useful tool for the investigation of the inflammatory microenvironment of NP. These results were obtained entirely *in vitro*, and any conclusions about the actual effects of MSCs in NP *in vivo* remain to be explored. However, our findings clearly demonstrate an immunoregulatory effect of MSCs on immune cells (especially T cells) derived from nasal tissue affected by polyposis. Finally, we hope that further studies will be performed in the search for an understanding of the mechanism of MSC activity in the context of NP inflammation.

## Figures and Tables

**Table 1:** 

Patients' comorbidities			
Identification	Asthma	Aspirin intolerance	Rhinitis
ECR	+	+	−
FFS	+	+	−
JFE	−	−	−
JFS	+	−	−
LD	+	+	+
MAL	−	−	−
MGN	+	−	+
MS	−	−	−
MFV	+	+	+
NEAP	+	−	−
RSC	+	−	+
RPS	+	+	−

## References

[1] Hastan D, Fokkens WJ, Bachert C (2011). Chronic rhinosinusitis in Europe—an underestimated disease. A GA^2^LEN study. *Allergy*.

[2] Pezato R, Świerczyńska-Krępa M, Niżankowska-Mogilnicka E, Derycke L, Bachert C, Pérez-Novo CA (2012). Role of imbalance of eicosanoid pathways and staphylococcal superantigens in chronic rhinosinusitis. *Allergy*.

[3] van Bruaene N, Pérez-Novo CA, Basinski TM (2008). T-cell regulation in chronic paranasal sinus disease. *Journal of Allergy and Clinical Immunology*.

[4] Zhang N, van Zele T, Perez-Novo C (2008). Different types of T-effector cells orchestrate mucosal inflammation in chronic sinus disease. *Journal of Allergy and Clinical Immunology*.

[5] van Bruaene N, Derycke L, Perez-Novo CA (2009). TGF-*β* signaling and collagen deposition in chronic rhinosinusitis. *Journal of Allergy and Clinical Immunology*.

[6] Balsalobre L, Pezato R, Perez-Novo C (2013). Epithelium and stroma from nasal polyp mucosa exhibits inverse expression of TGF-*β*1 as compared with healthy nasal mucosa. *Journal of Otolaryngology—Head & Neck Surgery*.

[7] Watelet JB, Bachert C, Claeys C, van Cauwenberge P (2004). Matrix metalloproteinases MMP-7, MMP-9 and their tissue inhibitor TIMP-1: expression in chronic sinusitis vs nasal polyposis. *Allergy*.

[8] Lee Y-M, Kim S-S, Kim H-A (2003). Eosinophil inflammation of nasal polyp tissue: relationships with matrix metalloproteinases, tissue inhibitor of metalloproteinase-1, and transforming growth factor-*β*1. *Journal of Korean Medical Science*.

[9] Sejima T, Holtappels G, Bachert C (2011). The expression of fibrinolytic components in chronic paranasal sinus disease. *American Journal of Rhinology and Allergy*.

[10] Pezato R, Voegels RL (2012). Why do we not find polyps in the lungs? Bronchial mucosa as a model in the treatment of polyposis. *Medical Hypotheses*.

[11] Bousquet J, Jacquot W, Vignola AM, Bachert C, van Cauwenberge P (2004). Allergic rhinitis: a disease remodeling the upper airways?. *Journal of Allergy and Clinical Immunology*.

[12] Holgate ST, Holloway J, Wilson S, Bucchieri F, Puddicombe S, Davies DE (2004). Epithelial-mesenchymal communication in the pathogenesis of chronic asthma. *Proceedings of the American Thoracic Society*.

[13] Wang L-F, Chien C-Y, Chiang F-Y, Chai C-Y, Tai C-F (2012). Corelationship between matrix metalloproteinase 2 and 9 expression and severity of chronic rhinosinusitis with nasal polyposis. *American Journal of Rhinology and Allergy*.

[14] Li X, Meng J, Qiao X (2010). Expression of TGF, matrix metalloproteinases, and tissue inhibitors in Chinese chronic rhinosinusitis. *Journal of Allergy and Clinical Immunology*.

[15] le Blanc K, Tammik L, Sundberg B, Haynesworth SE, Ringdén O (2003). Mesenchymal stem cells inhibit and stimulate mixed lymphocyte cultures and mitogenic responses independently of the major histocompatibility complex. *Scandinavian Journal of Immunology*.

[16] Devine SM, Cobbs C, Jennings M, Bartholomew A, Hoffman R (2003). Mesenchymal stem cells distribute to a wide range of tissues following systemic infusion into nonhuman primates. *Blood*.

[17] Bassi ÊJ, de Almeida DC, Moraes-Vieira PMM, Câmara NOS (2012). Exploring the role of soluble factors associated with immune regulatory properties of mesenchymal stem cells. *Stem Cell Reviews and Reports*.

[18] Parekkadan B, Milwid JM (2010). Mesenchymal stem cells as therapeutics. *Annual Review of Biomedical Engineering*.

[19] Silva FS, Almeida PN, Rettore JV (2012). Toward personalized cell therapies by using stem cells: seven relevant topics for safety and success in stem cell therapy. *Journal of Biomedicine and Biotechnology*.

[20] le Blanc K, Ringdén O (2007). Immunomodulation by mesenchymal stem cells and clinical experience. *Journal of Internal Medicine*.

[21] Nauta AJ, Fibbe WE (2007). Immunomodulatory properties of mesenchymal stromal cells. *Blood*.

[22] Di Nicola M, Carlo-Stella C, Magni M (2002). Human bone marrow stromal cells suppress T-lymphocyte proliferation induced by cellular or nonspecific mitogenic stimuli. *Blood*.

[23] Fokkens WJ, Lund V, Mullol J (2012). European position paper on rhinosinusitis and nasal polyps 2012. *Rhinology*.

[24] Lennon DP, Caplan AI (2006). Isolation of human marrow-derived mesenchymal stem cells. *Experimental Hematology*.

[25] Pittenger MF, Mackay AM, Beck SC (1999). Multilineage potential of adult human mesenchymal stem cells. *Science*.

[26] Galindo LT, Filippo TR, Semedo P (2011). Mesenchymal stem cell therapy modulates the inflammatory response in experimental traumatic brain injury. *Neurology Research International*.

[27] Semedo P, Donizetti-Oliveira C, Burgos-Silva M (2010). Bone marrow mononuclear cells attenuate fibrosis development after severe acute kidney injury. *Laboratory Investigation*.

[28] Chen S-L, Fang W-W, Ye F (2004). Effect on left ventricular function of intracoronary transplantation of autologous bone marrow mesenchymal stem cell in patients with acute myocardial infarction. *American Journal of Cardiology*.

[29] Horwitz EM, Gordon PL, Koo WKK (2002). Isolated allogeneic bone marrow-derived mesenchymal cells engraft and stimulate growth in children with osteogenesis imperfecta: implications for cell therapy of bone. *Proceedings of the National Academy of Sciences of the United States of America*.

[30] Rojas M, Xu J, Woods CR (2005). Bone marrow-derived mesenchymal stem cells in repair of the injured lung. *American Journal of Respiratory Cell and Molecular Biology*.

[31] Fierabracci A, del Fattore A, Luciano R, Muraca M, Teti A, Muraca M (2013). Recent advances in mesenchymal stem cell immunomodulation. The role of microvisicles. *Cell Transplantation*.

[32] Bassi ÊJ, Moraes-Vieira PM, Moreira-Sá CS (2012). Immune regulatory properties of allogeneic adipose-derived mesenchymal stem cells in the treatment of experimental autoimmune diabetes. *Diabetes*.

[33] Dai F, Shi D, He W (2006). hCTLA4-gene modified human bone marrow-derived mesenchymal stem cells as allogeneic seed cells in bone tissue engineering. *Tissue Engineering*.

[34] Huang J, Guo J, Beigi F (2014). HASF is a stem cell paracrine factor that activates PKC epsilon mediated cytoprotection. *Journal of Molecular and Cellular Cardiology*.

[35] Tomic S, Djokic J, Vasilijic S (2011). Immunomodulatory properties of mesenchymal stem cells derived from dental pulp and dental follicle are susceptible to activation by toll-like receptor agonists. *Stem Cells and Development*.

[36] English K, Ryan JM, Tobin L, Murphy MJ, Barry FP, Mahon BP (2009). Cell contact, prostaglandin E_2_ and transforming growth factor beta 1 play non-redundant roles in human mesenchymal stem cell induction of CD4^+^CD25^High^forkhead box P3^+^ regulatory T cells. *Clinical and Experimental Immunology*.

[37] Patel SA, Meyer JR, Greco SJ, Corcoran KE, Bryan M, Rameshwar P (2010). Mesenchymal stem cells protect breast cancer cells through regulatory T cells: role of mesenchymal stem cell-derived TGF-*β*. *Journal of Immunology*.

[38] Nemeth K, Keane-Myers A, Brown JM (2010). Bone marrow stromal cells use TGF-*β* to suppress allergic responses in a mouse model of ragweed-induced asthma. *Proceedings of the National Academy of Sciences of the United States of America*.

[39] Yang YC, Zhang N, van Crombruggen K, Hu GH, Hong SL, Bachert C (2012). Transforming growth factor-beta1 in inflammatory airway disease: a key for understanding inflammation and remodeling. *Allergy*.

[40] Pérez-Novo CA, Claeys C, van Cauwenberge P, Bachert C (2006). Expression of eicosanoid receptors subtypes and eosinophilic inflammation: implication on chronic rhinosinusitis. *Respiratory Research*.

[41] English K, Barry FP, Field-Corbett CP, Mahon BP (2007). IFN-*γ* and TNF-*α* differentially regulate immunomodulation by murine mesenchymal stem cells. *Immunology Letters*.

[42] Chen K, Wang D, Du WT (2010). Human umbilical cord mesenchymal stem cells hUC-MSCs exert immunosuppressive activities through a PGE2-dependent mechanism. *Clinical Immunology*.

[43] Németh K, Leelahavanichkul A, Yuen PST (2009). Bone marrow stromal cells attenuate sepsis via prostaglandin E 2-dependent reprogramming of host macrophages to increase their interleukin-10 production. *Nature Medicine*.

[44] Djouad F, Charbonnier L-M, Bouffi C (2007). Mesenchymal stem cells inhibit the differentiation of dendritic cells through an interleukin-6-dependent mechanism. *Stem Cells*.

[45] Xu G, Zhang Y, Zhang L, Ren G, Shi Y (2007). The role of IL-6 in inhibition of lymphocyte apoptosis by mesenchymal stem cells. *Biochemical and Biophysical Research Communications*.

[46] Kishimoto T (2006). Interleukin-6: discovery of a pleiotropic cytokine. *Arthritis Research and Therapy*.

[47] Liu X-J, Zhang J-F, Sun B (2009). Reciprocal effect of mesenchymal stem cell on experimental autoimmune encephalomyelitis is mediated by transforming growth factor-*β* and interleukin-6. *Clinical and Experimental Immunology*.

[48] Huang L, Baban B, Johnson BA, Mellor AL (2010). Dendritic cells, indoleamine 2,3 dioxygenase and acquired immune privilege. *International Reviews of Immunology*.

[49] Honkanen T, Luukkainen A, Lehtonen M (2011). Indoleamine 2,3-dioxygenase expression is associated with chronic rhinosinusitis with nasal polyps and antrochoanal polyps. *Rhinology*.

[50] Ge W, Jiang J, Arp J, Liu W, Garcia B, Wang H (2010). Regulatory T-cell generation and kidney allograft tolerance induced by mesenchymal stem cells associated with indoleamine 2,3-dioxygenase expression. *Transplantation*.

[51] Aggarwal S, Pittenger MF (2005). Human mesenchymal stem cells modulate allogeneic immune cell responses. *Blood*.

